# Angiotensin Receptor Blocker and Neprilysin Inhibitor Suppresses Cardiac Dysfunction by Accelerating Myocardial Angiogenesis in Apolipoprotein E-Knockout Mice Fed a High-Fat Diet

**DOI:** 10.1155/2021/9916789

**Published:** 2021-08-04

**Authors:** Yasunori Suematsu, Kohei Tashiro, Hidetaka Morita, Akihito Ideishi, Takashi Kuwano, Shin-ichiro Miura

**Affiliations:** ^1^Department of Cardiology, Fukuoka University School of Medicine, Fukuoka, Japan; ^2^Department of Cardiology, Fukuoka University Nishijin Hospital, Fukuoka, Japan

## Abstract

**Materials and Methods:**

Male apolipoprotein E-knockout mice fed a high-fat diet were divided into control (CTL), valsartan (30 mg/kg) (VAL), sacubitril (30 mg/kg) (SAC), and valsartan plus sacubitril (30 mg/kg each) (VAL/SAC) groups after 4 weeks of prefeeding and were subsequently treated for 12 weeks.

**Results:**

The VAL/SAC group exhibited significantly higher serum brain natriuretic peptide levels; more subtle changes in left ventricular systolic diameter, fractional shortening, and ejection fraction, and significantly higher expression levels of natriuretic peptide precursor B and markers of angiogenesis, including clusters of differentiation 34, vascular endothelial growth factor A, and monocyte chemotactic protein 1, than the CTL group.

**Conclusions:**

Valsartan plus sacubitril preserved left ventricular systolic function in apolipoprotein E-knockout mice fed a high-fat diet. This result suggests that myocardial angiogenic factors induced by ARNI might provide cardioprotective effects.

## 1. Introduction

Obesity [1], hypertension [2], diabetes mellitus [3], and metabolic syndrome [4] can cause cardiac hypertrophy, leading to heart failure [5, 6]. Myocardial angiogenesis is necessary for maintaining cardiac systolic function during cardiac hypertrophy [7]. Under overload conditions, cardiomyocytes become hypertrophic and myocardial angiogenesis accelerates in response to an increased demand for oxygen. However, sustained cardiac hypertrophy causes maladaptation, cardiac remodeling, and heart failure [7]. Evidence shows that myocardial angiogenesis can help prevent heart failure progression, and therapeutic angiogenesis is an important issue in the field of cardiovascular disease [8–10]. Nonetheless, optimal treatment protocols have yet to be established.

Neprilysin, also known as neutral endopeptidase, inactivates natriuretic peptides by cleaving a variety of peptide bonds [11]. Therefore, the valsartan/sacubitril combination serves as an angiotensin receptor blocker and neprilysin inhibitor (ARNI) [12], a type of medication that increases natriuretic peptide availability. The PARADIGM-HF clinical trials showed that the valsartan/sacubitril combination exhibited better cardioprotective effects against heart failure with reduced left ventricular ejection fraction (HFrEF) than angiotensin-converting enzyme inhibitors [13]. Moreover, the US and European guidelines for HF management recommend valsartan/sacubitril as first-line therapy for HFrEF [14, 15]. Furthermore, clinical trials have shown that valsartan/sacubitril promoted better reduction in secondary functional mitral regurgitation than valsartan (PRIME) [16], with another a clinical trial investigating the effects of valsartan/sacubitril on ventricular remodeling (i.e., PROVE-HF) currently ongoing [17]. Basic studies confirmed the pleiotropic effects of ARNI. Accordingly, our previous studies reported that ARNI exhibited antifibrotic cardioprotective effects against diabetic HFrEF [18], promoted antihypertrophic cardioprotective effects [19], improved renal function during chronic kidney disease [20], and suppressed aldosterone synthesis [21], and ARNI also affects pulmonary hypertension [22], endothelial dysfunction [23], and atherosclerotic plaque formation [24]. However, no study has yet investigated the effects of ARNI on myocardial angiogenesis in an early-stage cardiac dysfunction model. Therefore, the present study sought to determine whether valsartan plus sacubitril administration could improve cardiac dysfunction in an animal model of early-stage cardiac dysfunction.

## 2. Materials and Methods

### 2.1. Experimental Protocol

All experimental protocols were approved by the Animal Care and Use Committee of Fukuoka University and conformed to the Guide for the Care and Use of Laboratory Animals of the Institute of Laboratory Animal Resources.

Apolipoprotein E-knockout mice were purchased from Charles River Laboratories Japan, Inc., Japan. Apolipoprotein E-knockout mice with or without a high-fat diet reportedly exhibited cardiac dysfunction [25–28]. To establish a model of early-stage cardiac dysfunction, 8-week-old male apolipoprotein E-knockout mice were started on a high-fat diet (week 4) containing 0.5% cholesterol and 17% coconut oil with a normal chow diet. The proportions of calories from protein, fat, and nitrogen-free extract were 16.8%, 43.0%, and 40.2%, respectively. After 4 weeks of prefeeding, the mice were divided into control (CTL), valsartan (30 mg/kg) (VAL), sacubitril (30 mg/kg) (SAC), and valsartan plus sacubitril (30 mg/kg each) (VAL/SAC) groups (week 0). Drugs were administered by mixed drinking water. Body weight and blood pressure were measured every 4 weeks (weeks 4, 0, 4, 8, and 12). Blood pressure was measured through a tail cuff-based MK-2000 device (Muromachi Kikai Co., Ltd., Tokyo, Japan). Echocardiography was performed using isoflurane (2%–3%) at weeks 0 and 12. The drug dosages relied on previous basic research using valsartan and sacubitril [18–20, 22, 23]. Previous studies reported that male apolipoprotein E-knockout mice exhibited cardiac endothelial–mesenchymal transition after 8 weeks on a high-fat diet, starting from 8 weeks of age [27], and 7.5-month-old male apolipoprotein E-knockout mice exhibited endothelial dysfunction [25]. Therefore, our study investigated 8-week-old apolipoprotein E-knockout mice until they were 6 months old, during which early-stage cardiac dysfunction would have occurred based on the previous reports [25, 27]. After 12 weeks of treatment, we measured serum brain natriuretic peptide (BNP) levels with a RayBio Mouse BNP Enzyme Immunoassay Kit (Catalog #: EIAM-BNP, RayBiotech, GA, USA); the expression of messenger ribonucleic acid (mRNA) in the left ventricle was measured using reverse transcription–polymerase chain reaction (RT–PCR), and Masson's trichrome staining and clusters of differentiation 34 (CD34) immunostaining were performed in the left ventricle.

### 2.2. Evaluation of Cardiac Function

Echocardiographic measurements were performed using NEMIO SSA-550A (Toshiba, Tokyo, Japan). From the short-axis two-dimensional view and M mode at the level of the papillary muscle, we measured heart rate, interventricular septum thickness diameter (IVSTd), left ventricular internal dimension in diastole (LVDd), left ventricular posterior wall thickness diameter (LVPWd), left ventricular internal dimension in systole (LVDs), left ventricular ejection fraction (LVEF), and left ventricular fractional shortening (LVFS).

### 2.3. Quantitative Reverse Transcription–Polymerase Chain Reaction Analysis

mRNA expression levels were quantified using RT–PCR as previously described [18]. We extracted total ribonucleic acid from the apex of the left ventricle using a RiboPure RNA Purification Kit (Life Technologies, Carlsbad, CA, USA). We produced complementary deoxyribonucleic acid using a ReverTra Ace® qPCR RT Kit (TOYOBO, Japan). We performed quantitative RT–PCR on a 7500 Fast Real-Time PCR System (Applied Biosystems) using a THUNDERBIRD® SYBR® qPCR Mix (TOYOBO, Japan). We investigated natriuretic peptide type A (NPPA), natriuretic peptide type B (NPPB), transforming growth factor-*β* (TGF-*β*), myosin heavy chain 7 (MyH7), CD34, vascular endothelial growth factor A (VEGFA), monocyte chemotactic protein 1 (MCP1), gene of sarcoplasmic/endoplasmic reticulum calcium adenosine triphosphatase 2 (ATP2a2), vascular cell adhesion molecule-1 (VCAM-1), *β* catenin, vascular endothelial-cadherin (VE-cadherin), nuclear factor-kappa B (NF-*κ*B) inhibitor alpha (Nfkbia), inhibitor of NF-*κ*B kinase subunit beta (Ikbkb), inhibitor of NF-*κ*B kinase regulatory subunit gamma (Ikbkg), lysosome-associated membrane glycoprotein 2 (Lamp2), phosphatase and tensin homolog deleted on chromosome 10-induced kinase 1 (Pink1), and *β* actin in the left ventricule (LV).  Table 1 lists the primers. Although we also investigated tumor necrosis factor *α*, interleukin-4, interleukin-6, interleukin-10, natriuretic peptide type C, plasminogen activator inhibitor 1, endothelial nitric oxide synthase, and endoplasmic reticulum oxidoreductin-1 in the LV, we could not analyze them due to low or undetectable expression. mRNA levels were expressed relative to mRNA levels of *β* actin, and the basal expression relative to that in the CTL group was considered to be 1.0.

### 2.4. Histological Analysis

We evaluated the quantity of myocardial fibrosis in Masson's trichrome-stained heart sections. Left ventricular tissues of the midlayer were fixed with 4% paraformaldehyde and stained with Masson's trichrome. The percentage of fibrotic area in the left ventricle was analyzed using the Image J software. To quantify myocardial angiogenesis, we stained fixed left ventricular tissues for immunohistochemical analysis of CD34. The percentage of CD34-positive cell area in the left ventricle was analyzed using the Image J software. Masson's trichrome staining and CD34 immunostaining were performed using Biopathology Institute Co. (Oita, Japan), and digital photographs were taken using a BZ-9000 series All-in-one Fluorescence Microscope (Keyence Japan, Osaka, Japan).

### 2.5. Statistical Analysis

All data analyses were performed using SAS (version 9.4, SAS Institute Inc., Cary, NC, USA) at Fukuoka University (Fukuoka, Japan), with a *p* value of <0.05 indicating statistical significance. Continuous variables were expressed as mean ± standard deviation. Group differences were analyzed using a one-way analysis of variance.

## 3. Results

### 3.1. Changes in Body Weight and Blood Pressure

**Figure 1:**
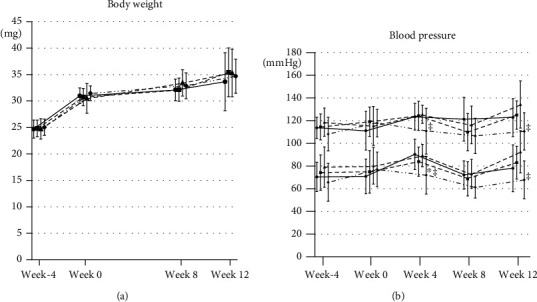
Changes in body weight and blood pressure. Changes in (a) body weight and (b) blood pressure, including systolic blood pressure and diastolic blood pressure in each group. CTL: control group; VAL: valsartan group; SAC: sacubitril group; VAL/SAC: valsartan plus sacubitril group. The round marker and solid line, square marker and dotted line, triangle marker and dashed line, and rhombus marker and chain line indicate CTL, VAL, SAC, and VAL/SAC. CTL (*n* = 8), VAL (*n* = 7), SAC (*n* = 8), and VAL/SAC (*n* = 8) were investigated. ∗ and ‡ indicate significant differences compared with CTL and SAC during the same week, respectively.

Figure 1 summarizes changes in body weight and blood pressure. Before prefeeding, the average baseline body weight and systolic blood pressure were 24.8 ± 1.6 g and 113.7 ± 12.2 mmHg, respectively. After 4 weeks of prefeeding, the average body weight and systolic blood pressure were 30.9 ± 1.9 g and 115.8 ± 14.6 mmHg, respectively. There were no significant differences between the groups. After 12 weeks of treatment, the average body weight and systolic blood pressure were 34.7 ± 4.3 g and 123.0 ± 18.3 mmHg, respectively. There were no significant differences between the treatment and CTL groups. None of the medications affected arterial blood pressure in the experimental animals.

### 3.2. Changes in Cardiac Functions

**Figure 2:**
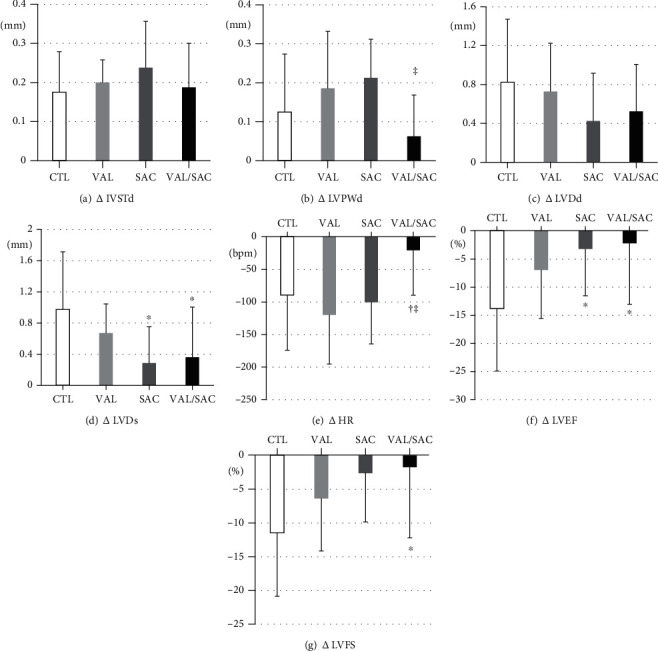
Changes in cardiac parameters via echocardiography after 12 weeks of treatment. Changes in (a) IVSTd, (b) LVPWd, (c) LVDd, (d) LVDs, (e) HR, (f) LVEF, and (g) LVFS in each group. IVSTd: interventricular septum thickness diameter; LVPWD: left ventricular posterior wall thickness diameter; LVDd: left ventricular internal dimension in diastole; LVDs: left ventricular internal dimension in systole; HR: heart rate; LVEF: left ventricular ejection fraction; LVFS: left ventricular fractional shortening; CTL: control group; VAL: valsartan group; SAC: sacubitril group; VAL/SAC: valsartan plus sacubitril group. CTL (*n* = 8), VAL (*n* = 7), SAC (*n* = 8), and VAL/SAC (*n* = 8) were investigated. ∗, †, and ‡ indicate significant differences compared with CTL, VAL, and SAC, respectively.

We investigated cardiac function using echocardiography at weeks 0 and 12 (Table 2).  Figure 2 details the changes in cardiac parameters after 12 weeks of treatment. In the CTL group, LVDs increased by 0.98 ± 0.74 mm, whereas LVEF and LVFS decreased by 13.9% ± 11.1% and 11.5% ± 9.4%, respectively, over 12 weeks. The VAL/SAC group showed a significantly smaller increase in LVDs (0.36 ± 0.64 mm; *p* = 0.04) and a significantly smaller decrease in LVEF (2.25% ± 10.9%; *p* = 0.03) and LVFS (1.8% ± 10.5%; *p* = 0.04) than the CTL group. The SAC group exhibited a significantly smaller increase in LVDs (0.29 ± 0.46 mm; *p* = 0.03) and a significantly smaller decrease in LVEF (3.3% ± 8.3%; *p* = 0.04) than the CTL group.

### 3.3. Serum BNP and mRNA Expression Levels of NPPA and NPPB in the LV

**Figure 3:**
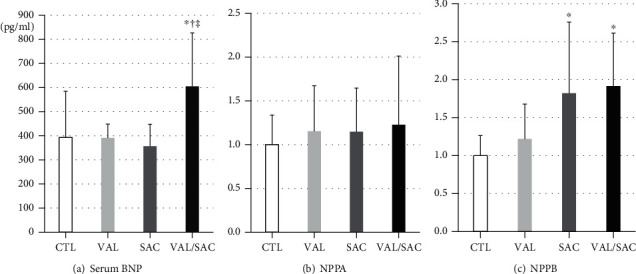
Serum BNP and mRNA expression levels of NPPA and NPPB in the LV. (a) Serum BNP levels and mRNA expression levels of (b) NPPA and (c) NPPB in each group. BNP: brain natriuretic peptide; NPPA: natriuretic peptide type A; NPPB: natriuretic peptide type B; CTL: control group; VAL: valsartan group; SAC: sacubitril group; VAL/SAC: valsartan plus sacubitril group. CTL (*n* = 8), VAL (*n* = 7), SAC (*n* = 8), and VAL/SAC (*n* = 7) were investigated. ∗, †, and ‡ indicate significant differences compared with CTL, VAL, and SAC, respectively.

Figure 3(a) presents serum BNP levels. After 12 weeks of treatment, the VAL/SAC group had significantly higher serum BNP levels than the CTL group (CTL: 393.2 ± 191.7 pg/mL and VAL/SAC: 605.2 ± 221.3 pg/mL, *p* = 0.01).

Figures 3(b) and 3(c) present detailed mRNA expression levels of NPPA and NPPB in the LV. The SAC and VAL/SAC groups had significantly higher expression levels of NPPB than the CTL group (SAC: 1.8 ± 0.9 times, *p* = 0.02 and VAL/SAC: 1.9 ± 0.7 times, *p* = 0.01) due to the effects of the neprilysin inhibitor. The neprilysin inhibitor did not increase NPPA expression in the LV since NPPA is mainly expressed in the atrium.

### 3.4. Cardiac Fibrosis and Hypertrophy in the LV

**Figure 4:**
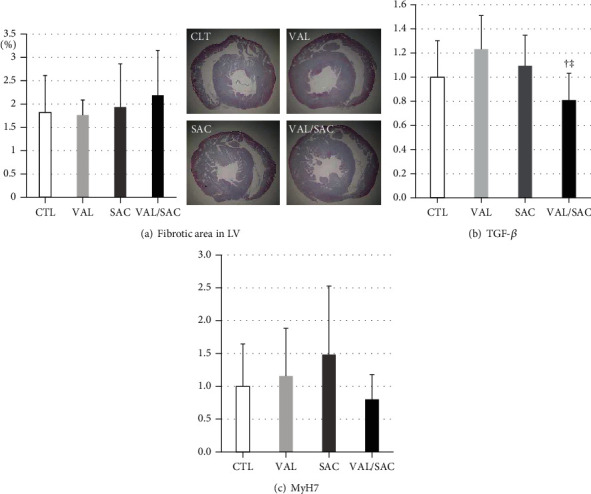
Cardiac fibrosis and hypertrophy in the LV. (a) Representative macrophotographs of the aorta with Masson's trichrome staining and quantification analysis for the percentage of fibrosis in each group. mRNA expression levels of (b) TGF-*β* and (c) MyH7 in each group are shown. TGF-*β*: transforming growth factor-beta; MyH7: myosin heavy chain 7; CTL: control group; VAL: valsartan group; SAC: sacubitril group; VAL/SAC: valsartan plus sacubitril group. CTL (*n* = 8), VAL (*n* = 7), SAC (*n* = 8), and VAL/SAC (*n* = 7) were investigated. † and ‡ indicate significant differences compared with VAL and SAC, respectively.

Considering our previous reports on the antifibrotic and hypertrophic effects of VAL/SAC [18, 19], we investigated cardiac fibrosis and hypertrophy in the LV. In this model, the CTL group showed only 1.8% ± 0.8% fibrosis following histological analysis, with no significant differences between the groups (Figure 4(a)). After investigating mRNA expression levels of TGF-*β* (a marker of fibrosis) and MyH7 (a marker of hypertrophy) in the LV, the treatment groups did not show significantly better improvement than the CTL group (Figures 4(b) and 4(c)).

### 3.5. Regulation of NF-*κ*B and Lysosome Activity

**Figure 5:**
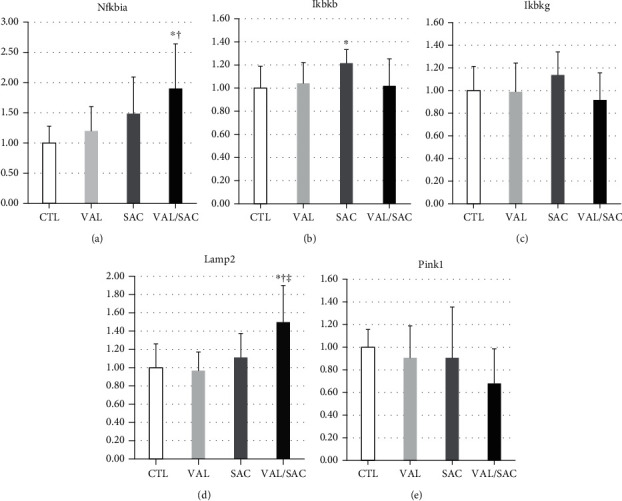
Regulation of NF-*κ*B and activity of mitochondria and lysosome. mRNA expression levels of (a) Nfkbia, (b) Ikbkb, (c) Ikbkg, (d) Lamp2, and (e) Pink1 in each group. NF-*κ*B: nuclear factor-kappa B; Nfkbia: NF-*κ*B inhibitor alpha; Ikbkb: an inhibitor of NF-*κ*B kinase subunit beta; Ikbkg: an inhibitor of NF-*κ*B kinase regulatory subunit gamma; Lamp2: lysosome-associated membrane glycoprotein 2; Pink1: phosphatase and tensin homolog deleted on chromosome 10-induced kinase 1; CTL: control group; VAL: valsartan group; SAC: sacubitril group; VAL/SAC: valsartan plus sacubitril group. CTL (*n* = 8), VAL (*n* = 7), SAC (*n* = 8), and VAL/SAC (*n* = 7) were investigated. ∗, †, and ‡ indicate significant differences compared with CTL, VAL, and SAC, respectively.

We investigated the regulation of NF-*κ*B, mitochondrial activity in mitophagy, and lysosomal activity in autophagy (Figures 5(a)–5(e)). Accordingly, the VAL/SAC group had greater mRNA expression of Nfkbia, an NF-*κ*B inhibitor, than the CTL group (Figure 5(a)). Moreover, the VAL/SAC group showed greater expression of Lamp2, which plays a critical role in autophagosome maturation, than the CTL group (Figure 5(d)).

### 3.6. Angiogenic Effect

**Figure 6:**
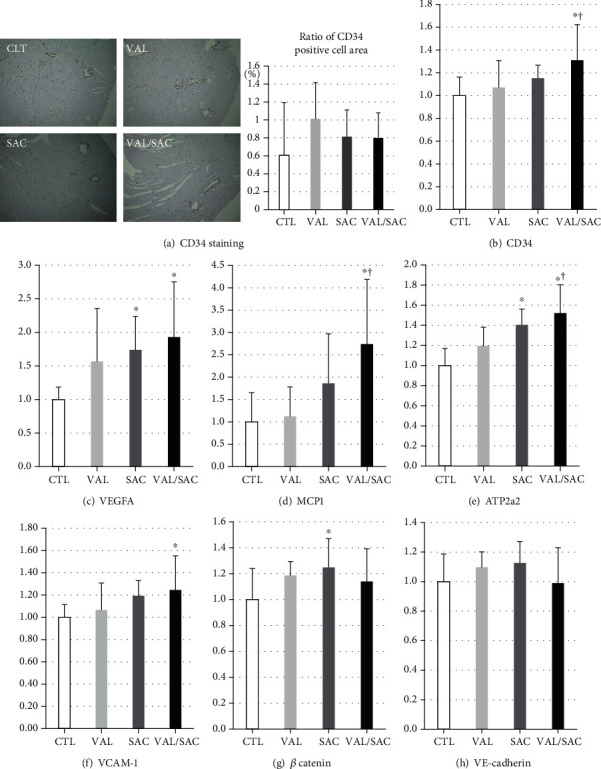
Myocardial angiogenesis. (a) Representative microphotographs of the left ventricle, with immunohistochemical staining for CD34, and a quantitative analysis for the percentage of the CD34-positive cell area in each group. mRNA expression levels of (b) CD34, (c) VEGFA, (d) MCP1, (e) ATP2a2, (f) VCAM-1, (g) *β* catenin, and (h) VE-cadherin in each group. CD34: clusters of differentiation 34; VEGFA: vascular endothelial growth factor A; MCP1: monocyte chemotactic protein 1; ATP2a2: gene name of sarcoplasmic/endoplasmic reticulum calcium adenosine triphosphatase 2; VCAM-1: vascular cell adhesion protein 1; VE-cadherin: vascular endothelial-cadherin; CTL: control group; VAL: valsartan group; SAC: sacubitril group; VAL/SAC: valsartan plus sacubitril group. CTL (*n* = 8), VAL (*n* = 7), SAC (*n* = 8), and VAL/SAC (*n* = 7) were investigated. ∗, †, and ‡ indicate significant differences compared with CTL, VAL, and SAC, respectively.

Figure 6(a) presents the results of histological analysis via CD34 immunostaining in the LV. Accordingly, there were no significant differences in the CD34-positive cell area in the LV between the groups. Figures 6(b)–6(h) show mRNA expression levels of markers of angiogenesis in the LV. The VAL/SAC group had significantly higher expression levels of CD34, VEGFA, MCP1, ATP2a2, and VCAM-1 but not *β* catenin or VE-cadherin (Figures 6(g) and 6(h)) than the CTL group (Figures 6(b)–6(f)).

## 4. Discussion

The present study showed that valsartan plus sacubitril increased myocardial angiogenic factors. The NF-*κ*B inhibitor preserved lysosomal activity and suppressed cardiac dysfunction in apolipoprotein E-knockout mice fed a high-fat diet independent of changes in cardiac fibrosis and hypertrophy in this model. Valsartan plus sacubitril demonstrated cardioprotective effects during early-stage cardiac dysfunction and ARNI might be useful for the primary prevention of heart failure via adaptation to the increase in oxygen demand.

Evidence showed that inhibition of the renin–angiotensin system promoted anti-inflammatory, antioxidant, and antifibrotic effects [29]. ARNI improved lymphatic system remodeling in a hypertrophic cardiomyopathy model [30] while decreasing oxidative stress and increasing adenosine triphosphate and Na+/K+-ATPase pump activity in ischemic reperfusion-induced arrhythmia [31]. However, no study has yet investigated the angiogenic effects of ARNI for adaptation to increased oxygen demand during cardiac hypertrophy.

Apolipoprotein E-knockout mice with or without a high-fat diet have been used to study atherosclerosis [32–36], plaque rupture [37], coronary artery disease [38], and cardiac dysfunction [25–28]. Studies have shown that myocardial hypertrophy due to peripheral vascular resistance [25], hypertension and endothelial dysfunction 26, myocardial fibrosis [27], and reduced cardiac functional reserve cause cardiac dysfunction in apolipoprotein E-knockout mice [28]. Therefore, cardiac hypertrophy can be considered a cause of cardiac dysfunction in apolipoprotein E-knockout mice.

The myocardial angiogenic effects of ARNI occur in response to the increased oxygen demand under cardiac hypertrophy. Our model showed that blood pressure increased slightly, and that pathological proportion of fibrosis and mRNA expression of plasminogen activator inhibitor 1, a marker of fibrosis in the LV, remained low. The present study found that valsartan plus sacubitril did not exert any antihypertensive, antihypertrophic, or antifibrotic effects, probably due to the mild pathology in the animal model used herein. This finding may be attributable to the animals' young ages, short experimental periods, mild high-fat diet, or their interaction. Moreover, only slight changes in cardiac dysfunction parameters were observed in this model, although valsartan plus sacubitril significantly suppressed the progression of cardiac dysfunction. The above results suggest that ARNI exerts cardioprotective effects in the early-stage of cardiac dysfunction, and that it can be useful for the primary prevention of heart failure onset during cardiac hypertrophy.

In this study, valsartan plus sacubitril increased serum BNP and mRNA expressions of NPPB in the LV, suppressed the dilation of LVDs, and preserved LVEF and LVFS. The PARADIGM-HF clinical trials showed that valsartan/sacubitril combination exerted cardioprotective effects against HFrEF [13]. Moreover, the US and European guidelines for HF management recommend valsartan/sacubitril combination as the first-line therapy for HFrEF [14, 15], with the present results being consistent with these recommendations. Our findings showed that one mechanism through which valsartan plus sacubitril exhibited cardioprotective effects was increased myocardial angiogenic factors. Based on our investigation, valsartan plus sacubitril increased mRNA expression levels of CD34, VEGFA, ATP2a2, and MCP1, all of which are myocardial angiogenic factors [39–42]. Autologous CD34-positive cell therapy for ischemic heart disease is associated with increased LVEF, exercise time, neovascularization, decreased angina, nitroglycerine use, heart failure, and mortality [43]. Meanwhile, ATP2a2 encodes sarcoplasmic/endoplasmic reticulum calcium adenosine triphosphatase 2 (SERCA2a). Cardiac SERCA2a has been associated with myocardial angiogenesis [40] and calcium recycling of the cardiac muscle [44], which is another therapeutic target for heart failure [45]. Increasing mRNA expression of ATP2a2 through valsartan plus sacubitril treatment might improve cardiac dysfunction through calcium recycling independent of myocardial angiogenesis. However, the present study did not investigate the detailed pathway of calcium recycling. Moreover, our findings showed that the valsartan plus sacubitril group had low mRNA expression levels of inflammatory markers, including tumor necrosis factor *α* and interleukin-6, but high mRNA expression levels of MCP1. After investigating anti-inflammatory markers, including interleukin-4 and interleukin-10, our findings showed that both had low mRNA expression levels. Our model showed that valsartan plus sacubitril did not have anti-inflammatory or inflammatory effects in the LV.

In the chronic phase, prolonged activation of NF-*κ*B is cytotoxic and promotes heart failure by triggering an inflammatory response [46]. NF-*κ*B is also a good regulator of cardiac hypertrophy [47]. Our findings showed that valsartan plus sacubitril increased the mRNA expression of Nfkbia, one of the main inhibitors of NF-*κ*B, suggesting that ARNI can regulate NF-*κ*B during early-stage cardiac dysfunction. Optimal autophagic activity is critical in the maintenance of cardiovascular homeostasis and function [48]. Autophagic or mitophagic flux in the cardiovascular system has been associated with the spontaneous development of cardiovascular disorders [49]. Lamp2 is a critical protein for autophagic flux. Danon disease, which occurs due to loss of function mutations in the Lamp2 gene, causes impaired mitophagy, facilitating mitochondrial damage [50]. The valsartan plus sacubitril group included herein showed high mRNA expression of Lamp2, which might be one of the effects of valsartan and sacubitril for early-stage cardiac dysfunction.

The present study has several limitations worth noting. First, older apolipoprotein E-knockout mice, a longer experimental period, and a higher-fat diet should have been used to investigate hypertension and cardiac fibrosis during cardiac hypertrophy. However, given the fact that our focus was on the angiogenic effects of valsartan plus sacubitril for early-stage cardiac dysfunction, our model can be deemed appropriate for this study. Second, our CD34 immunostaining analysis showed no valsartan plus sacubitril-induced enhancement of myocardial angiogenesis. Although ARNI in another animal model [51] and natriuretic peptide [52–54] reportedly exhibited angiogenic effects, some studies show that natriuretic peptide suppresses angiogenesis [55, 56]. However, the present study found considerably low mRNA levels of endothelial markers in the LV, including endothelial nitric oxide synthase, endoplasmic reticulum oxidoreductin-1, and NPPC. Such discrepant outcomes need to be carefully considered in future studies, together with the use of other animal models in the investigation of myocardial angiogenesis and endothelial markers in the heart.

## 5. Conclusions

The present study showed that valsartan plus sacubitril preserved left ventricular systolic function in apolipoprotein E-knockout mice fed a high-fat diet. The ARNI-induced myocardial angiogenic factors possibly explain its cardioprotective effects.

## Figures and Tables

**Table 1:** Primer sequences used in quantitative RT–PCR.

Gene		Sequence (5′ to 3′)
NPPA	Forward	GGGGGTAGGATTGACAGGAT
	Reverse	ACACACCACAAGGGCTTAGG
NPPB	Forward	TCCTAGCCAGTCTCCAGAGC
	Reverse	CCTTGGTCCTTCAAGAGCTG
TGF-*β*	Forward	GCTTCTAGTGCTGACGCCCG
	Reverse	GACTGGCGAGCCTTAGTTTG
MyH7	Forward	GAGGAGAGGGCGGACATC
	Reverse	GGAGCTGGGTAGCACAAGAG
CD34	Forward	GACAACATGTGGTGGCTGAC
	Reverse	AGCTGAAGGCAGCATGAAGT
VEGFA	Forward	CAGGCTGCTGTAACGATGAA
	Reverse	TATGTGGCTGGCTTTGGTGAG
MCP1	Forward	AGCACCAGCCAACTCTCACT
	Reverse	GGCGTTAACTGCATCTGGCT
ATP2a2	Forward	TACTGACCCTGTCCCTGACC
	Reverse	CACCACCACTCCCATAGCTT
VCAM-1	Forward	ACAGACAGTCCCCTCAATGG
	Reverse	ACCTCCACCTGGGTTCTCTT
*β* catenin	Forward	GTGCAATTCCTGAGCTGACA
	Reverse	CTTAAAGATGGCCAGCAAGC
VE-cadherin	Forward	ACCTTTCAGATGCAGCGACT
	Reverse	TGGCACACCATCATCTTGTTTT
Nfkbia	Forward	TCGCTCTTGTTGAAATGTGG
	Reverse	CTCTCGGGTAGCATCTGGAG
Ikbkb	Forward	GAGCTGTCCTTACCCTGCTG
	Reverse	TGCTGCAGAACGATGTTTTC
Ikbkg	Forward	TGAAGAAATGCCAACAGCAG
	Reverse	CTAAAGCTTGCCGATCCTTG
Lamp2	Forward	ATTTGGCTAATGGCTCAGCTT
	Reverse	GAAAGCACCTGCTCTTTGTTG
Pink1	Forward	TTGAGGAGCAGACTCCCAGT
	Reverse	AGTCCCACTCCACAAGGATG
*β* actin	Forward	CCACACCCGCCACCAGTTCG
	Reverse	TACAGCCCGGGGAGCATCGT

NPPA: natriuretic peptide type A; NPPB: natriuretic peptide type B; TGF-*β*: transforming growth factor-beta; MyH7: myosin heavy chain 7; CD34: clusters of differentiation 34; VEGFA: vascular endothelial growth factor A; MCP1: monocyte chemotactic protein 1; ATP2a2: gene name of sarcoplasmic/endoplasmic reticulum calcium adenosine triphosphatase 2; VCAM-1: vascular cell adhesion protein 1; VE-cadherin: vascular endothelial-cadherin; Nfkbia: nuclear factor-kappa B (NF-*κ*B) inhibitor alpha; Ikbkb: inhibitor of NF-*κ*B kinase subunit beta; Ikbkg: inhibitor of NF-*κ*B kinase regulatory subunit gamma; Lamp2: lysosome-associated membrane glycoprotein 2; Pink1: phosphatase and tensin homolog deleted on chromosome 10-induced kinase 1.

**Table 2:** Cardiac functions by echocardiography in pre and posttreatment.

	CTL	VAL	SAC	VAL/SAC
Week 0				
HR	641 ± 76	636 ± 56	619 ± 84	596 ± 69
IVSTd	0.53 ± 0.09	0.54 ± 0.05	0.54 ± 0.12	0.53 ± 0.07
LVPWd	0.64 ± 0.13	0.66 ± 0.13	0.63 ± 0.05	0.68 ± 0.07
LVDd	3.6 ± 0.5	3.5 ± 0.4	3.6 ± 0.5	3.8 ± 0.4
LVDs	2.0 ± 0.4	1.9 ± 0.4	2.2 ± 0.3	2.3 ± 0.4
LVEF	81.6 ± 4.8	81.8 ± 5.3	79.4 ± 3.7	78.9 ± 5.3
LVFS	44.8 ± 5.1	44.8 ± 5.3	42.3 ± 3.8	41.9 ± 5.3
Week 12				
HR	551 ± 42	513 ± 45	518 ± 43	575 ± 48^†‡^
IVSTd	0.70 ± 0.05	0.74 ± 0.05	0.78 ± 0.10^∗^	0.71 ± 0.06
LVPWd	0.76 ± 0.09	0.87 ± 0.11^∗^	0.84 ± 0.11	0.74 ± 0.07^†‡^
LVDd	4.4 ± 0.3	4.2 ± 0.3	4.0 ± 0.5	4.3 ± 0.2
LVDs	2.9 ± 0.4	2.5 ± 0.3	2.4 ± 0.5^∗^	2.6 ± 0.4
LVEF	67.8 ± 8.2	76.0 ± 7.2^∗^	76.1 ± 7.5^∗^	76.6 ± 6.7^∗^
LVFS	33.3 ± 6.1	39.6 ± 6.4	39.6 ± 6.3	40.1 ± 6.1^∗^

CTL: control group; VAL: valsartan group; SAC: sacubitril group; VAL/SAC: valsartan plus sacubitril group; HR: heart rate; IVSTd: interventricular septum thickness diameter; LVPWd: left ventricular posterior wall thickness diameter; LVDd: left ventricular internal dimension in diastole; LVDs: left ventricular internal dimension in systole; LVEF: left ventricular ejection fraction; LVFS: left ventricular fractional shortening. ∗, †, and ‡ show significant differences compared to CTL, VAL, and SAC, respectively.

## Data Availability

No data were used to support this study.
